# Mucoadhesive Mesoporous Silica Particles as Versatile Carriers for Doxorubicin Delivery in Cancer Therapy

**DOI:** 10.3390/ijms241914687

**Published:** 2023-09-28

**Authors:** Mirela-Fernanda Zaltariov, Bianca-Iulia Ciubotaru, Alina Ghilan, Dragos Peptanariu, Maria Ignat, Mihail Iacob, Nicoleta Vornicu, Maria Cazacu

**Affiliations:** 1 Department of Inorganic Polymers, “Petru Poni” Institute of Macromolecular Chemistry, Aleea Gr. Ghica Voda 41 A, 700487 Iasi, Romania; ciubotaru.bianca@icmpp.ro (B.-I.C.); ignat.maria@icmpp.ro (M.I.); iacob.mihai@icmpp.ro (M.I.); 2 Department of Natural Polymers, Bioactive and Biocompatible Materials, “Petru Poni” Institute of Macromolecular Chemistry, Aleea Gr. Ghica Voda 41 A, 700487 Iasi, Romania; diaconu.alina@icmpp.ro; 3 Centre of Advanced Research in Bionanoconjugates and Biopolymers, “Petru Poni” Institute of Macromolecular Chemistry, Aleea Gr. Ghica Voda 41 A, 700487 Iasi, Romania; peptanariu.dragos@icmpp.ro; 4 Department of Chemistry, “Alexandru Ioan Cuza” University of Iasi, 700506 Iasi, Romania; 5 Metropolitan Center of Research T.A.B.O.R, The Metropolitanate of Moldavia and Bukovina, 700497 Iasi, Romania; cmctaboriasi@yahoo.com

**Keywords:** mesoporous silica particles, doxorubicin carrier, cytotoxicity, mucoadhesion test, antimicrobial activity

## Abstract

Due to their structural, morphological, and behavioral characteristics (e.g., large volume and adjustable pore size, wide functionalization possibilities, excellent biocompatibility, stability, and controlled biodegradation, the ability to protect cargoes against premature release and unwanted degradation), mesoporous silica particles (MSPs) are emerging as a promising diagnostic and delivery platform with a key role in the development of next-generation theranostics, nanovaccines, and formulations. In this study, MSPs with customized characteristics in-lab prepared were fully characterized and used as carriers for doxorubicin (DOX). The drug loading capacity and the release profile were evaluated in media with different pH values, mimicking the body conditions. The release data were fitted to Higuchi, Korsmeyer–Peppas, and Peppas–Sahlin kinetic models to evaluate the release constant and the mechanism. The in vitro behavior of functionalized silica particles showed an enhanced cytotoxicity on human breast cancer (MCF-7) cells. Bio- and mucoadhesion on different substrates (synthetic cellulose membrane and porcine tissue mucosa)) and antimicrobial activity were successfully assessed, proving the ability of the OH- or the organically modified MSPs to act as antimicrobial and mucoadhesive platforms for drug delivery systems with synergistic effects.

## 1. Introduction

Silica nanoparticles (SNPs) are studied as promising cancer-targeting carriers. Due to their low toxicity, biocompatibility, and intrinsic characteristics (large surface area, adjustable pore size, and multifunctionality), mesoporous silica (MS) can be used to load a variety of therapeutics (nucleotides, drugs, and imaging agents) and to release them under controlled conditions (acid pH mimicking the extracellular environment of cancer tissue, overexpressed enzymes and biomolecules, magnetic field, ultrasound, etc.) [1,2,3]. Clinical trials conducted on core–shell silica-based nanoplatforms with a size of 7 nm loaded with Cy5 dye, bioconjugated with polyethylene glycol (PEG), and radiolabeled RGD peptide showed long-term effectiveness for imaging cancer cells using positron emission tomography (PET). Cornell dots are currently being investigated as imaging detection agents in metastases, brain, or prostate cancer [4]. Recent biomedical research advances involving MSNPs may constitute the basis of future diagnostic approaches and individualized therapy [1,2]. MS particles are suitable for designing complex multifunctional theranostic nanosystems for cancer therapy and imaging (Magnetic Resonance Imaging (MRI), Photoacoustic (PA), Physical Therapy (PT), Near-Infrared Fluorescence (NIRF), Positron Emission Tomography (PET), Single-Photon Emission Computed Tomography (SPECT), Computed Tomography (CT), sonography, Multispectral Optoacoustic Tomography (MSOT)), gene therapy, and editing [3]. By tuning their pore size, stimuli-responsive ability, and co-delivering of more than one bioactive agent, MS particles show a huge potential in the therapeutics of multidrug resistance (MDR) cells. So far, MS particles also have been developed for targeted gene and protein delivery and tissue engineering. Recently, MSNPs have been investigated as top candidates intended for photoactivated therapy and multiple combination therapy as diagnostic and therapeutic platforms. Their characteristics such as vast specific surface area allowing high loading efficiency of photothermal agents (PTA) or photosensitizers (PS), the variable morphology adjusting the drug release and cell uptake behavior, and the simple functionalization to ensure a targeted release of PTA/PS make them suitable for the fight against cancer [5]. It also has been reported that gold-SNPs undergo clinical trials for the plasmonic photothermal therapy (PPT) treatment of atherosclerosis [4]. In addition, MS and MS-based hybrid NPs were reported as suitable nanoplatforms in nanodynamic therapy [6]. The use of SNPs in drug delivery has proven advantageous from the point of view of the limitations induced by the stability and solubility of drugs and their adverse side effects, poor release, and MDR for a high therapeutic efficacy [7]. SNPs offer the ability to overcome the MDR by limiting the intrinsic (physical barriers of entry), extrinsic (tumor surrounding microenvironment), or drug-induced mechanisms by tuning their shell pore size and stimuli-responsive behavior, co-delivering mixed therapeutics through a specific endosomal process (enhanced permeability and retention effect (EPR)), avoiding the rapid clearance and the recognition by reticuloendothelial system (RES) [8,9]. In combination with photothermal therapy (PTT), MSNPs coating polydopamine (PDA) were reported as hard templates for hollow mesoporous carbon nanoparticles (MCN), which demonstrated an effective suppression of MDR in cancer cells [10]. Despite the major potential for drug delivery systems, this application is still insufficiently developed to allow commercialization, being a key direction mainly for the pharmaceutical field, some improvements being necessary for therapeutic efficiency [8].

The previous particularities can be controlled by surface functionalization of the particles. Usually, there are two general methods for surface functionalization: in situ co-condensation using different templates (cetyltrimethylammonium bromide (CTAB)—or ionic liquids) and silica precursors and post-functionalization procedures by grafting the active surface silanol groups, allowing the insertion of various organic groups or organic shielding polymeric or pH/thermal responsive coatings. The intrinsic silica surface can be tailored using different additives during the synthetic process [11,12]. The surface modification of MS for biological applications has a higher impact on the interaction with cells, the uptake and accumulation of NPs by the cells and tissues, etc. [13,14].

Besides the functionality, the particle size will decide the mechanism of the intracellular uptake and cell internalization: pinocytosis (particle size up to 50 nm), clathrin- and caveolin-mediated endocytosis (particle size between 150 nm and 200 nm), micropinocytosis, and phagocytosis (particle size from 250 nm to 3 μm) [15]. However, an optimum size of 50–250 nm appears to be most favorable to ensure efficient cell uptake and long blood circulation time, avoiding particle clearance through the kidney and allowing a high passive tumor accumulation by the EPR effect. The particle shape can also affect the internalization, cell viability, adhesion, migration, and distribution in the organism. Thus, it was proven that the long rod-like shaped NPs exhibited a higher rate of accumulation in the largest quantity. The reduced size of MSNPs could also be responsible for their toxicity by accumulation in the liver and spleen [8,16].

The compatibility and the safety of the administration of MSNPs are required conditions. Some immune responses may occur depending on their concentration, size, surface charge, shape, and loaded drugs. Optimal effects mean an efficient drug release, reducing the frequently needed dosages, by generation of a “depot-effect” [17] together with minimal toxicity and a targeted intracellular release of the drugs [18]. Although silica is commonly assumed to be a safe material, the degradation studies on MSNPs do not provide a complete profile of their specific accumulation, biodegradation, and biodistribution, hindering clinical advancements in MSNPs [19].

In this paper, the preparation of surface-modified silica particles with organic (methyl) and/or functional (amino and hydroxyl) groups for DOX transport and release is reported. The MS (M1-M5) and DOX-loaded (D1-D5) MS particles were characterized by Fourier-Transform Infrared (FT-IR) spectroscopy, N_2_ sorption, Dynamic Light Scattering (DLS), Zeta potential, and Transmission Electron Microscopy (TEM). The drug loading capacity and the release profile were evaluated in media with different pH values, mimicking the body conditions. The release profile was fitted to various kinetic models: Higuchi, Korsmeyer–Peppas, and Peppas–Sahlin, to evaluate the release constant and the mechanism of release. The obtained silica particles were also investigated for their bio- and mucoadhesivity (on synthetic cellulose membrane and porcine tissue mucosa) and their antimicrobial and cytotoxicity activities on several species of fungi and bacteria and normal/cancer cells (Human Gingival Fibroblast (HGF), breast cancer (MCF-7), cervical cancer (HeLa)). The results indicated that the functionalities of silica can significantly influence the drug loading capacity, the release behavior, the cytotoxicity on normal and cancer cells, as well as some other specific biologic properties: antimicrobial and mucoadhesive properties on mucosa tissues.

## 2. Results

### 2.1. FT-IR Spectroscopy of MS and DOX-Loaded MSPs

**Figure 1:**
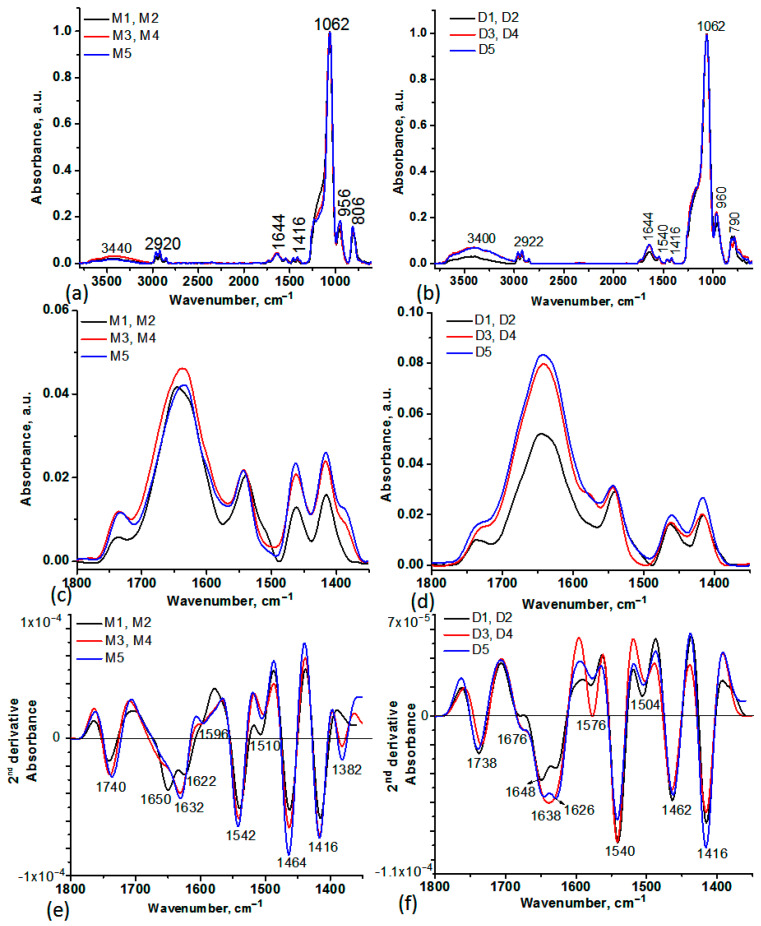
FT-IR spectra in absorbance of MS before (M1-M5) (**a**) and after (D1-D5) (**b**) DOX encapsulation. Spectra details in the 1800–1400 cm^−1^ spectral range of MS before (M1-M5) (**c**) and after (D1-D5) (**d**) DOX encapsulation, where “M” denoted the MS samples before DOX loading and “D” denoted the MS samples after DOX loading. The 2nd derivative of the IR spectra in the 1800–1400 cm^−1^ spectral range of MS before (M1-M5) (**e**) and after (D1-D5) (**f**) DOX encapsulation. The negative bands in the 2nd derivative spectra correspond to the maxima of absorbance spectra in the same spectral region.

The IR spectra of the MS before and after DOX encapsulation revealed the presence of the characteristic vibrations assigned to the Si-O stretches at 1062 cm^−1^ and 960 cm^−1^, N-H and O-H stretches at 3400–3440 cm^−1^, C-H asymmetric and symmetric stretches at 2920-2855 cm^−1^, and C-H deformation and bending at 1464 and 1416 cm^−1^ [20]. The weak and broad bands in 1800–1500 cm^−1^ highlighted by the second derivative of the spectra are characteristic of overtones modes of Si-O at 1740 cm^−1^, N-H deformation vibrations (M1, M2), and O-H functionalities in M3, M4 (Figure 1a,c,e) [21,22]. These bands are blueshifted by 4–6 cm^−1^ in D1, D2, D3, and D4 samples confirming the interactions of silica functionalities with DOX (Figure 1b,d,f). The 2nd derivative of the IR spectra of DOX-loaded MS samples evidenced negative peaks at 1676 and 1576 cm^−1^ (Figure 1f) corresponding to the maxima absorptions found in the 1800–1300 cm^−1^ spectral range (Figure 1d). These bands are specific to C=O stretching vibration, N-H deformation, and C=C ring stretch in DOX [23].

**Figure 2:**
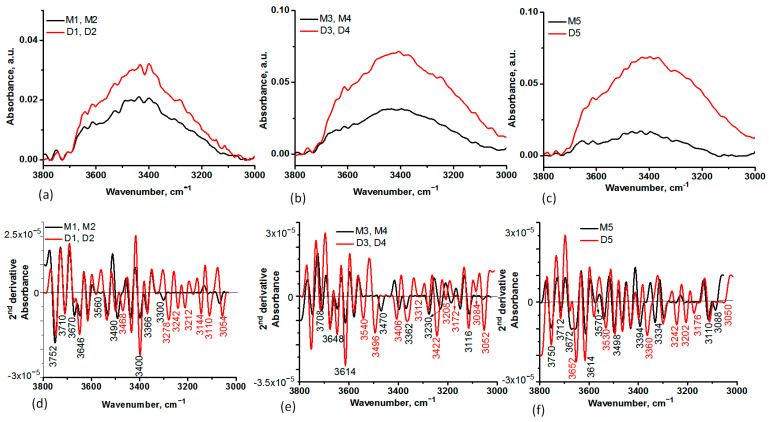
Spectra details in the 3800-3000 cm^−1^ spectral range of MS before and after DOX encapsulation: M1, M2, D1, D2 (**a**), M3, M4, D3, D4 (**b**), and M5, D5 (**c**), where “M” denoted the MS samples before DOX loading and “D” denoted the MS samples after DOX loading. The 2nd derivative of the IR spectra in the 3800–3000 cm^−1^ spectral range of MS before and after DOX encapsulation: M1, M2, D1, D2 (**d**), M3, M4, D3, D4 (**e**), and M5, D5 (**f**). The negative bands in the 2nd derivative spectra correspond to the maxima of absorbance spectra in the same spectral region. The marked negative peaks in red revealed the spectra modification after DOX encapsulation.

**Figure 3:**
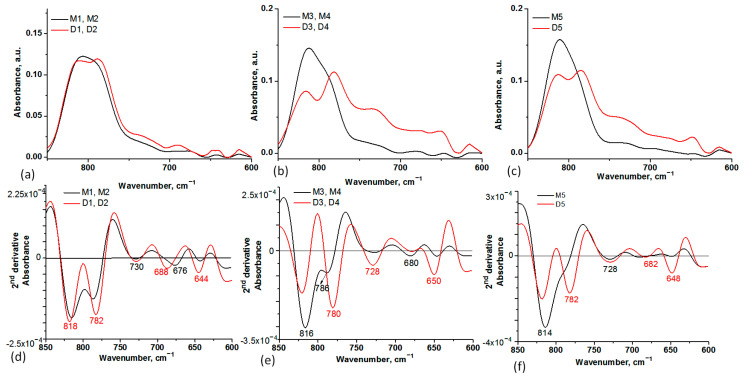
Spectra details in the 850–600 cm^−1^ spectral range of MS before and after DOX encapsulation: M1, M2, D1, D2 (**a**), M3, M4, D3, D4 (**b**), and M5, D5 (**c**), where “M” denoted the MS samples before DOX loading and “D” denoted the MS samples after DOX loading. The 2nd derivative of the IR spectra in the 850–600 cm^−1^ spectral range of MS before and after DOX encapsulation: M1, M2, D1, D2 (**d**), M3, M4, D3, D4 (**e**), and M5, D5 (**f**). The negative bands in the 2nd derivative spectra correspond to the maxima of absorbance spectra in the same spectral region. The marked negative peaks in red revealed the spectra modification after DOX encapsulation.

The interaction of MS with DOX was also highlighted by the spectral details in the 3800–3000 cm^−1^ and 850–600 cm^−1^, observed with the second derivative of the spectra (Figure 2 and Figure 3). In the 3800–300 cm^−1^ spectral range, the presence of DOX is confirmed by the maxima at 3242–3054 cm^−1^ characteristic for N-H stretches and C-H stretching in the aromatic ring. The characteristic stretches of N-H and O-H in M1, M2, M3, and M4, respectively, changed their initial position after DOX encapsulation, confirming the binding of DOX at the surface of the silica (Figure 2).

In the 850–600 cm^−1^ spectral range, the 2nd derivative of the spectra showed negative peaks at 782 cm^−1^, 682 cm^−1^, and 644 cm^−1^ characteristic of absorption maxima found in DOX spectrum (C=H bend, C=C ring bend) [23] (Figure 3).

### 2.2. DLS and Zeta Potential Analyses

The surface modification of the MSPs and the particle size before and after DOX encapsulation were evaluated by Zeta potential and DLS analyses (Table 1 and Figures S1 and S2).

DLS analyzes the intensity variations in the scattered light to calculate the size of the particles subjected to Brownian motions, expressed as the hydrodynamic diameter. Various parameters, such as the refractive indices of the materials and the media, are also considered to calculate the DLS number-weighted distributions. Therefore, whenever these parameters differ from the theoretically estimated ones, like particle sphericity and homogeneity, some differences in particle size can occur [24,25]. The average diameter values of MS particles as a 5% suspension in water, estimated by DLS, were between 825 and 1280 nm, 712 and 955 nm, and 396 and 531 nm, with polydispersity indices of 0.216, 0.546, and 0.478 for M1, M2, and M3, respectively. In the cases of M4 and M5, the presence, although in low percentages (13.5% and 7.5%, respectively) of a second population with a smaller diameter was highlighted, which led to slightly higher values of the polydispersity (0.615 and 0.572, respectively) (Figure S1).

The particle size after DOX encapsulation showed a similar evolution as M1-M5 samples (Figure S2). The average diameter of DOX-loaded MS particles varied between 458 nm (D5) and712 nm (D1), with higher PDI values being observed for D2 (0.614), D4 (0.611), and D5 (0.603), where a second population of particles with a diameter of about 100 nm was evidenced.

The Zeta potential value of M1 increased after DOX encapsulation, while for M2-M5 samples, even after DOX loading, the surface charge of MSPs remains negatively charged. These values are also dependent on the nanomaterial properties and the solution pH, ionic strength, concentrations, and other additives [26].

### 2.3. TEM Morphology of the MSPs before and after Loading of DOX

For morphological and size investigation of the MS particles, before and after DOX loading, TEM images were also acquired (Figures S3 and S4). One can observe that DOX loading in all MS particle samples does not change the initial morphology of the particles. M1 and M2 exhibited a rod-shape morphology with large particle size > 500 nm in length and a diameter of 150–200 nm (Figures S3 and S4a,b), while M3, M4, and M5 exhibited spherical and/or ellipsoid particles with a 200–400 nm diameter size (Figures S3 and S4c–e).

### 2.4. Release of DOX from MS Samples

**Figure 4:**
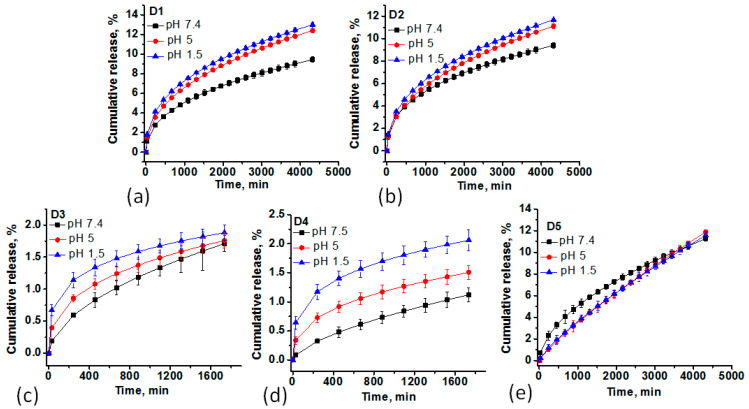
The CR (%) of DOX during 72 h at pH 1.5, 5, and 7.4 for D1 (**a**), D2 (**b**), D3 (**c**), D4 (**d**), and D5 (**e**) fitted with Krosmeyer–Peppas kinetic model. All data are presented as the mean with SD (standard deviation) from at least three independent experiments. Statistical evaluation of the variance differences was conducted using ANOVA at significance levels of *p* < 0.05.

The in vitro release studies of DOX from MS samples were performed at 37 °C in buffer solutions at pH 1.5, 5, and 7.4, corresponding to the normal and extracellular tumor environments, respectively, for 72 h. Figure 4 displays the experimental data of the CR (%) of DOX from D1-D5 samples. One can observe a limited drug release for all samples regardless of the pH value, the lowest release being observed for D3 and D4 samples, only 2.2–2.5% after 72 h. Within 24 h, a maximum release of 8% was found for D1, D2, and D5 under low acidity conditions (pH 1.5 and pH 5).

To investigate the release kinetics, the obtained release profiles were fitted to three kinetic models (Higuchi, Korsmeyer–Peppas, and Peppas–Sahlin), and the data are shown in Table 2. The release data were best fitted with the Korsmeyer–Peppas model (Equation (5)) and Peppas–Sahlin (Equation (6)) at all pH values (Table 2). The release tests of D3 and D4 showed a very slow release after the 1800 min, so the sustained release after this time was ignored in the kinetics evaluation. The first-order model was not suitable because the MS samples were not soluble in aqueous media. On the other hand, the Higuchi model better describes the release of the drugs from the matrix, while the Korsmeyer–Peppas is better used for drug release from polymeric systems controlled by diffusion mechanisms [22]. Particularly, the Peppas–Sahlin model takes into account the effects of the Fickian diffusion and case II transport—relaxation (erosion) contribution. One can observe that *K*_2_ < *K*_1_ supports that the release of DOX is mainly controlled by diffusion [23].

Based on the literature data, it can be said that the conjugation of mesoporous silica with different proteins, lipids, or other liposomal and polyelectrolyte derivatives would have a positive impact, especially on the release of DOX [24,27,28,29,30,31,32,33,34,35,36,37,38,39,40,41,42]. The cumulative release could increase up to 90% in some cases [43] and can vary widely depending on the functionalities of silica or post-functionalization with different pH-responsive polymeric compounds.

### 2.5. The Evaluation of Cytotoxicity of DOX-Loaded MS Samples

The cytotoxicity of MS samples, before and after DOX encapsulation, was investigated on three cell lines: normal cell line (HGF) and two cancer cell lines (MCF-7 and HeLa). The investigation of the MS compatibility on a normal cell line at a concentration of 30 ± 0.2 μg/mL revealed moderate biocompatibility for all samples (Figure S5a). The relative cytotoxicity on normal cells of 80–90% could be explained by the silica functionality, involving a strong interaction with the cells followed by its internalization promoting cell death [44].

**Figure 5:**
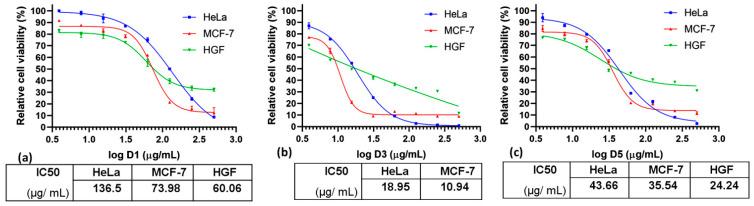
Cytotoxicity of the DOX-loaded MS samples: (**a**) D1, (**b**) D3, and (**c**) D5 on HeLa, MCF-7, and HGF cell lines. Relative IC_50_ values were determined by non-linear regression variable slope with four parameters using the Graphpad Prism software. All data are presented as the mean with standard deviation from three independent experiments. Statistical evaluation of differences was conducted using ANOVA at significance levels of *p* < 0.05.

The DOX-loaded MS cytotoxicity at a concentration of 30 ± 0.2 μg/mL revealed a lower viability on HGF cells and a higher viability on HeLa (90%) and MCF-7 (80%) for the D1 sample (Figure S5b). The IC_50_ values (Table 3) of D1 were 136.5 ± 0.2 μg/mL on HeLa and 73.98 ± 0.1 μg/mL on MCF-7, which correspond to a concentration of encapsulated DOX of 0.90 ± 0.2 μg/mL on HeLa and 0.49 ± 0.3 μg/mL on MCF-7, respectively (Figure 5a).

For the D3 sample having a higher DOX concentration encapsulated, a stronger cytotoxicity was observed. The viability of the HGF cells is 40%, as compared with the M3 sample (viability of 60%), with a high selectivity on cancer cells, especially for MCF-7 (viability of 10%), even if the IC_50_ value was not reached in the examined range of concentrations for HGF cell line (Figure 5b). The effect, in this case, is explained by DOX-released cytotoxicity cumulated with that of the silica (M3). The stronger effect of this sample than D1 is due to the higher loading capacity of DOX (32.7 ± 0.3 μg/mg) as compared with D1 with 6.7 ± 0.2 μg/mg DOX. The D5 sample also exhibited a stronger effect on HGF cells at this concentration (30 ± 0.2 μg/mL) than the initial silica (M5). The effect is also due to the DOX release, the loading capacity of this sample being 36 ± 0.2 μg/mg. A moderate cytotoxic effect was also observed on cancer cells, the viability being 60–70% on MCF-7 and HeLa cells, respectively (Figure S5c), the IC50 values on the cancer cells being 43.66 ± 0.2 μg/mL for HeLa and 35.54 ± 0.21 μg/mL for MCF-7 (Table 3) corresponding to 1.56 ± 0.1 μg/mL and 1.27 ± 0.1 μg/mL of DOX, respectively (Figure 5c).

At higher concentrations, all samples showed increased cytotoxicity, especially on MCF-7 cells. The selectivity indexes (Table 3) also revealed a high selectivity of D1 and D5 samples on MCF-7. The effect is almost equal for these samples, regardless of the amount of DOX encapsulated, suggesting that other mechanisms can be involved in cell death, such as uptake into tumor cells promoted by functionalities or accumulation of large aggregates into the cell culture [24].

### 2.6. Evaluation of Bio- and Mucoadhesion of the Functionalized MS

Taking into account that a large number of drugs are administered on different mucosal surfaces, we investigated the bio- and mucoadhesive properties of the MS particles.

**Figure 6:**
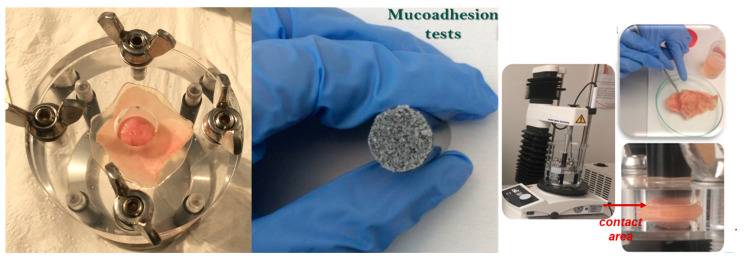
Mucoadhesion test images registered during the preparation of the tissue mucosa and evaluation of MS by TA.XT Plus texture equipment.

Bioadhesion of the MS samples was tested at pH 7.4 at 37 °C. Mucoadhesion tests (Figure 6) were performed on different mucosa tissues: stomach, small and large intestine, and colon in a physiologically simulated environment with a temperature of 37 °C and PBS media with different pH values varying from 1.5 to 8.5, according to the tissue region.

The in vitro method for estimation of the bioadhesion and mucoadhesion of a material with a substrate (either synthetic or biological) consists of the determination of the detachment force and the work of adhesion. The work of adhesion is the area under the force–distance curve obtained during the detachment process. An adhesion process mainly occurs in two stages: the contact stage (wetting/swollen stage) and the consolidation stage (strengthening process of interaction through chain entanglements and secondary bonds).

**Figure 7:**
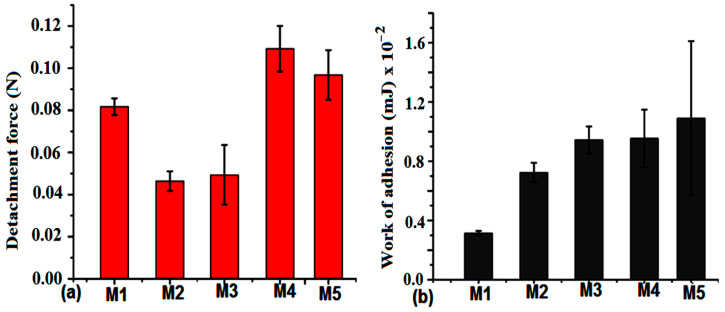
The detachment force (**a**) and the work of adhesion (**b**) of the MS samples with a synthetic membrane substrate. All data are shown as the mean with standard deviation from three independent experiments. Statistical evaluation of the variance differences was conducted using ANOVA at significance levels of *p* < 0.05.

The bioadhesivity of MS samples with a synthetic cellulose membrane revealed a strong interaction of M3 and M4 samples facilitated by the presence of hydroxyl groups on the surface. The detachment force was higher for M1 and M4 samples (Figure 7a), while the work of adhesion was higher for M3 and M4 samples (Figure 7b). The presence of the amino and hydroxyl groups explains the adhesion phenomenon for these samples. The variability of the detachment force within the same functionalities can be due to the different concentrations of these groups in contact with the membrane.

The mucoadhesion values on the stomach and small intestine mucosa were evaluated at different pH values, contributing to different values of the detachment force and work of adhesion. Thus, higher values were found at higher pH values corresponding to the small intestine medium (Table 4). The amino and hydroxyl functional groups proved to be responsible for the increased values of MS samples. This also can be explained by the amount of mucus present in the stomach compared to the intestine tissue.

The detachment force on colon tissue was twice as high as that found for large intestine mucosa (Table 5), while the work of adhesion was higher mainly for the M5 sample. The variability of these values was observed on colon tissue, depending on the mucus layer on the surface. In these segments, almost all samples have similar values, comparable with those found for stomach mucosa.

### 2.7. The Antimicrobial Screening of the Functionalized MS

MS samples were screened for their in vitro antifungal and antibacterial activity against pure cultures of four fungi species (*Aspergillus fumigatus*, *Fusarium*, *Penicillium frequentans*) and both Gram-negative (*Pseudomonas* sp.) and Gram-positive bacteria (*Bacillus* sp.). According to these assays, M1 and M3 exhibited suitable antifungal activity with a minimum inhibitory concentration (MIC) value of 0.38 ± 0.17 μg/mL and 0.75 ± 0.09 μg/mL, respectively, in comparison with the reference compound caspafungin (0.2 ± 0.02 μg/mL). The same compounds exhibited suitable antibacterial activity with MIC of 2 ± 0.02 μg/mL (M1) and, respectively, 3 ± 1.12 μg/mL (M3). The latter has a similar activity as the reference compound Kanamycin (MIC = 3 ± 0.02 μg/mL) (Table 5).

## 3. Discussion

Doxorubicin (DOX) hydrochloride is one of the most used chemotherapeutic drugs in the treatment of various human cancers. Despite its efficacy in the treatment of breast cancer, sarcomas, and hematological cancers, DOX exhibits major cumulative dose-dependent cardiotoxicity. Its therapeutical efficiency is further compromised due to the drug’s poor stability in biological media and low membrane permeability [45], so its encapsulation in MS could increase its bioavailability and accumulation in tumors, avoiding systemic toxicity.

FT-IR spectroscopy was used to investigate the spectral changes that occurred in the MS particles as a result of DOX loading or interactions established between them (Figure 1, Figure 2 and Figure 3). To establish the changes that occurred in silica particle size and surface after DOX loading, DLS and Zeta potential analyses were also performed. A few differences were observed, especially for the M1 and M2 samples and their corresponding DOX-loaded samples, D1 and D2, indicating that DOX majorly interacts with the functional groups on the surface of the particle, less than it is entrapped in the mesopores (Table 2). This observation is also supported by the lower DOX EE% for these samples. A few differences between MS before and after DOX encapsulation can be observed in the case of M3, M4, and M5 silica particles. Because of the positive charge of DOX in a neutral aqueous medium, silica samples possessing negatively charged silanol groups on the surface will entrap DOX mainly by electrostatic interactions. EE% was five times higher for these samples. The DLS measurements showed the presence of a broad size distribution of MS particles before and after DOX encapsulation (Figures S1 and S2), suggesting that some level of agglomeration may have occurred [46]. DLS can be an effective method to investigate the aggregation behavior of the particles. However, the particle size estimated by DLS is also dependent on the concentration, the scattering angle, the shape anisotropy and polydispersity of the particles, the surface roughness, and electroviscous effects [47]. The size of the particle will influence the mechanism of the cellular uptake. This mechanism implies phagocytosis by macrophages in the case of microscale particles, while for the small particles (200–300 nm), specific pathways of the endocytosis process are involved: interaction with the cell surface, followed by invagination, pinching off, and forming vesicles. The surface charge and particle shape will also regulate the internalization process, which can be cell-type-dependent. The cellular uptake will increase when a higher value of the negative charge is emphasized. It was demonstrated that spherical particles reached a total internalization in a shorter time than rod-shaped ones [46]. Despite the close correlation between particle size and antitumor effect, there are some differences related to tumor models, route of administration, etc. Thus, in subcutaneous tumors, the specific pore cutoff size varies from 200 nm to 1.2 μm. Moreover, it was proved that the structure and the environment of tumors have a contradictory effect related to the particle size; large particles tend to be more retained in tumor tissue than smaller ones, but the smaller ones possess better penetration ability in tumors. These challenges have been used to design some strategies for the development of particles with intelligent tunable size, such as aggregation or shrinkage or reversible size-changing approaches [48].

The Zeta potential values (Table 1) of the MS particles before and after DOX loading revealed a greater or lesser decrease mainly in dependence on the localization of DOX (in the pores or at the surface of the particles). DOX exhibits amphiphilic properties and can be attached by its antracycline ring by hydrophobic interactions or by electrostatic interactions at the surface. The pKa of DOX is 9.53, so at pH 7.4 in PBS medium, where the amino and hydroxylic groups are positively charged, DOX is bound through electrostatic interactions of the positively charged protonated amino groups o M1/M2 with negatively charged silanol groups in the pores and between the protonated silanol groups on the surface of M3/M4 and carbonyl groups from DOX.

The morphology of MS particles evaluated by TEM revealed differences between the samples (Figures S3 and S4), confirming that the nanoparticles obtained by the “surfactant micelle-templated” method are mainly influenced by the type of surfactant, the type and concentration of the silica precursor, the pH of the reagent medium, temperature, stirring speed and reaction time, and the presence of other additives. Different interactions, such as electrostatic, hydrophobic, or hydrogen bonds between the organo-alkoxysilane and the surfactant, can compete to shape the morphology of the nanoparticles. All the silica samples were obtained under similar conditions, the only difference being the silica precursor used, which waste traethoxysilane alone or partially substituted with a trimethoxysilane containing aminopropyl or methyl groups. Having different molecular masses to respect the molar ratio, the amounts of precursors differ, which changes the mass ratio between them and the surfactant. In addition, the amino, hydroxyl, and methyl groups will influence the pH of the reaction medium in a different way [49,50]. Previous studies have shown that by changing the precursor or its concentration, the particle morphology can take various shapes (spheres, tubes, rods) of different dimensions [51].

The mechanism of DOX release from MS samples was estimated based on the diffusion coefficients (Table 2), which showed a close value to 0.43 for M1–M4 samples supporting a Fickian diffusion (the release dependent on the diffusion mechanism), while for D5, the value of n is 0.43 < n < 0.83, supporting an anomalous transport (non-Fickian diffusion), a release dependent on swelling, degradation, or relaxation of the matrix. MSNPs demonstrated high stability under physiological conditions so that the erosion is limited only to the outermost layers. The higher constant rate, K, was found for samples D1 and D2, where DOX was bonded mainly on the surface. These results suggest that the aggregation of DOX in the mesopores through electrostatic interactions could block them, limiting the release, even at lower pH values. The lower values can be assigned in this case only to the DOX release from the surface [52]. This behavior can be an advantage in preventing the DOX release before the MS particles enter the cancer cell. Even if the DOX amount is low, some studies confirm that prolonged exposure to the drug at a modest concentration will be more beneficial than a quick initial burst. Moreover, a pronounced release is often accompanied by high cardiotoxicity and, in many cases, by a limited absorption of the drug into the circulation due to the efflux transporters [53].

The same release profile of DOX was observed for hybrid and hollow nanogels, where the lower DOX release was attributed to the effect of cavities, which trapped the DOX molecules, reducing the release rate [22]. However, a series of decisive factors have to be considered before the biomedical applications, which mainly are dictated by the surface functionalities and particle size: positive/negative charge of particles, aggregation behavior, interaction with nano-biointerfaces, plasma protein adsorption to surface, and intracellular trafficking of particles [54]. Long-term biocompatibility of MSNPs was also found to be dependent on the concentration, the particle size, the cell type, and the administration route. Thus, at higher concentrations (250–500 mg/mL), the macrophage cells were more sensible than the cancer cells, while the particle size lower than 360 nm induced no pathologies in major organs when administered in vivo [55]. A lack of toxicity was found at subcutaneous administration, even at high doses, while peritoneal or intravenous administration routes are accompanied by major toxic effects at higher concentrations [56]. The D1-D5 samples exhibited selectivity on MCF-7 cells (Figure 5, Table 3), the same cytotoxic effect of the samples suggesting that other mechanisms are involved in cell death. The functionality of the particles, as well as the accumulation of the samples through aggregation into the cell culture, can be considered in this case [24].

The functionalization of the particles can also influence the muco-penetration ability of the particles. This feature has been intensively studied, especially as an alternative to improve drug bioavailability when orally administered. There are multiple strategies to address the mucoadhesivity of micro-/nanoparticles, such as the use of mucin glycoproteins to produce nanosystems for enhanced drug delivery. A novel category of nanosystemsare known as mucosomes, a promising class of drug delivery systems, described as glycosylated mucoadhesive particles containing mucin with low immunogenicity, inert over the coagulation cascade and no cytotoxic effect on culture cell [57]. Other classes of polymers have also been evaluated to develop mucoadhesive particles. Thus, thiolated silica grafted with poly(2-oxazoline)-based derivatives with side chain length variation proved changes in the diffusion and penetration processes, in dependence on the polymer architecture [58]. MS particles functionalized with thiol groups exhibited a significantly higher mucoadhesivity on the urothelium than the corresponding hydroxyl- and amino derivatives, mainly due to the formation of disulfide bonds between thiol groups and cysteine-rich mucus constituents. These particles showed sustained drug delivery ability in bladder cancer therapy [59].

The study of the mucoadhesion parameters of the silica particles (Table 4) is also essential to appreciate the safety profile when they are locally administered because these particles can accumulate, becoming toxic to organisms. So, the elimination and the degradation process depend on their size, surface functionalization, charge, etc. It was proven that larger particles are eliminated mainly by the gastrointestinal tract, while the smaller ones are by the urinary tract. The biodegradation process of these particles mainly occurs by hydrolysis, silanols being the major by-products. Intestinal mucoadhesion is also important for the pH-dependent release and adsorption of some silica-loading drugs when administered orally or intraperitoneal [60,61]. While tumor malignancy and metastatic spread rank among the major causes of cancer death, a significant number of immunocompromised cancer patients die due to chemotherapy-associated infections. The clinical use of most conventional therapies is limited either by insufficient therapeutic drug concentrations at the target tissue or by severe toxic effects of the drug on healthy tissues. More people die only from infections and cardiovascular diseases. Therefore, the fight against cancer will continue to be the greatest challenging issue for medicinal and biochemical research. In this context, it is necessary to design nanocarriers able to protect, transport, and deliver bioactive agents in a controlled manner once they reach the target site. So the development of new nanosystems, namely “nanobiotics”, as a new alternative to fighting against infections in combination with cancer therapy is a great challenge.

MSNPs are considered a versatile class for many biomedical applications, especially in antimicrobial therapeutics, owing to their intrinsic functionalities, controllable pore size, and shape. The mechanism of their antimicrobial activity can occur by the physical damage of cell membrane, *reactive oxygen species* (ROS) production, endocytosis, etc. Also, the possibility of these particles developing antimicrobial resistance is lower, so they can be applied successfully, mainly in combined therapeutic systems. Recently, a silica fluorescent derivative known as Cornell dots received FDA approval for application in cancer diagnosis. SNPs proved a high efficacy in drug delivery of antibiotics and anticancer drugs. Also, it was tested for antibiofilm coating of implants, proving a higher activity against *C. albicans* and *S. oralis*. Silver-containing silica proved a higher efficiency against *E. coli* and *S. aureus*, with no associated immunotoxicity effects [62].

Amine functionalization of MSNPs proved to be effective as a target agent to recognize bacteria strains. The charged amino groups on the surface of MSNPs allow attractive electrostatic interactions with the bacteria or biofilm (with a negative density of charge), meanwhile with the release of the anticancer drugs, improving the efficiency [63].

The higher antimicrobial activity of M1 silica particles (Table 5) is due to the higher basicity of the amino groups, which can also affect cell viability [64]. The cell membranes of microorganisms consist of an abundance of negative charges on the surface due to the presence of anionic liposaccharides and phosphatidylglycerol, thus being explained the higher interaction of amino-functionalized silica particles. Besides the influence of the silica functionality on their biocidal activity, their hydrophobicity must be considered so that both hydrophobic and donor–acceptor interactions with the membrane lipid bilayers are involved in the antimicrobial mechanism [65,66].

## 4. Materials and Methods

### 4.1. Materials

Tetraethyl orthosilicate, TEOS (≥99%), (3-aminopropyl)triethoxysilane, APTES (≥98%), and triethoxymethylsilane, MTES (lane, were acquired from Sigma-Aldrich, Darmstadt, Germany and used as received. Hexadecyltrimethylammonium bromide (or CTAB) (>99%) was purchased from Acros Organics (Geel, Belgium). DOX, as an active pharmaceutical principle, Doxorubicin hydrochloride 2 mg/mL solution for infusion (commercially available) was achieved from Accord Healthcare (London, UK). Saline phosphate buffer (PBS) solutions: with pH 1.5 (prepared by mixing 44.6 mL citric acid 0.1 M with 5.4 mL dibasic sodium phosphate 0.2 M, adjusted with HCl 0.2 M in 1000 mL distilled water), with pH 5 (prepared by mixing 24.3 mL citric acid 0.1 M and 25.7 mL dibasic sodium phosphate 0.2 M in 1000 mL distilled water) and with pH 7.4 (prepared by dissolution of 8 g sodium chloride, 0.2 g potassium chloride, 1.44 g disodium phosphate and 0.24 g monopotassium phosphate in 1000 mL distilled water). Cells for cytotoxicity studies were purchased from PromoCell (Heidelberg, Germany) (HGF) and CLS Cell Lines Service GmbH, Eppelheim, Germany (HeLa and MCF-7). aMEM (Eagle’s Minimal Essential Alpha Medium), DMEM (Dulbecco’s Modified Eagle Medium) and antibiotic solution (with 1% mixture of Penicillin/Streptomycin/Amphotericin B, 10K/10/25 μg/mL) were purchased from Lonza. Fetal Bovine Serum (FBS) was purchased from Biochrom GmbH (Berlin, Germany). The CellTiter-Glo^®^ kit 2.0 assay, test for cell proliferation and evaluation of toxicity, was purchased from Promega Fisher Scientific (London, UK), while Tryple was from Thermo Fisher Scientific (London, UK). PBS was purchased from Invitrogen.

### 4.2. Preparation of MSPs with Various Surface Groups (M1-M5)

**Figure 8:**
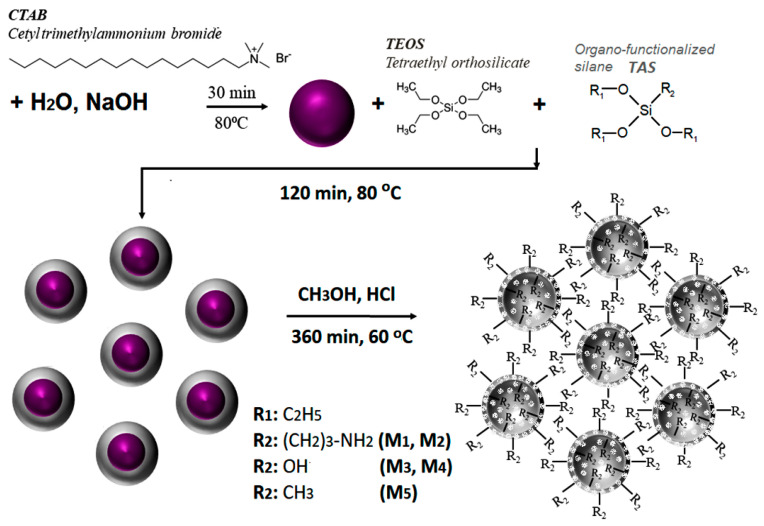
Schematic representation of the preparation protocol of in situ surface organo-modified and/or functionalized silica particles. The purple ball indicates the micelle from which the surfactant forms in water and which further constitutes the template for the formation of mesoporous silica.

MSPs in situ modified with organic and/or functional groups were prepared by surfactant micelle-templated one [51], consisting in co-condensing TEOS with trialkoxysilanes (TAS) containing the desired organic groups (i.e., -(CH_2_)_3_-NH_2_ or -CH_3_) or TEOS homocondensation, all in aqueous basic medium (NaOH), and in the presence of CTAB surfactant in low concentration (Figure 8).

The working protocol consists ofthe formation of a mixture of surfactant (CTAB) (2 g) and NaOH solution (7 mL, 8% in water) in distilled water (480 mL), heating this mixture for 30 min at 80 °C with stirring, followed by the addition of TEOS (10 mL, 44.78 mmol) and TAS (5.75 mmol) and the resumption of heating for a further 2 h under the same conditions. The formed silica is isolated by filtration, washed with water and methanol, and dried at 80 °C under vacuum for 18 h. To release/activate the pores, the material is subjected to extraction in a mixture of concentrated hydrochloric acid and methanol (1mL:100 mL for 1 g of dry material) at 60 °C for 6 h, after which the surfactant-free product isolated by hot filtration (60 °C) is washed with water and methanol, and dried again in vacuo. TAS was APTES leading to M1 and M2 silicas with NH_2_ groups on the internal and external surfaces, or MTES, leading to M5 silica, while the M3 and M4 samples were obtained by homocondensation TEOS generator of Si-OH groups on the surface. Incorporation of amino and hydroxyl moieties in the structure of silica can facilitate DOX loading via electrostatic interactions, thus improving the stability of the drug delivery system, while in the silica functionalized with methyl groups (M5), the drug encapsulation is greatly influenced by the hydrophobic interactions. Table 6 shows the pore size and the BET surface estimated by N_2_ sorption–desorption isotherms (Figure S6).

### 4.3. DOX Loading in MS (Samples D1-D5)

**Figure 9:**
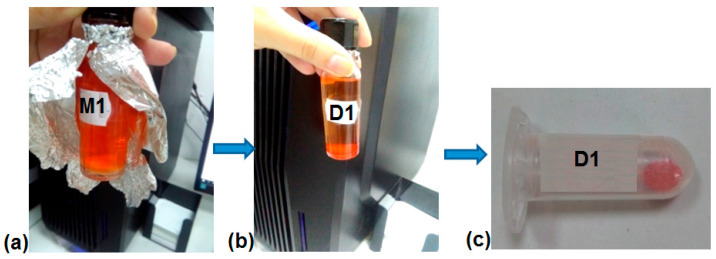
The color changes during the loading of DOX: (**a**) M1 in DOX solution, (**b**) D1 in DOX solution, (**c**) D1 in dried state.

The loading experiments were performed at room temperature (RT), at pH 7.4. About 10 mg MS particles were dispersed in 4 mL DOX solution 0.1% in PBS with pH 7.4. The mixture was ultrasonicated for 30 s; after that, it was stirred overnight in a rotator shaker at RT in dark conditions (Figure 9a). The next day, the loaded silica was centrifuged (Figure 9b), and the supernatant was separated and investigated by UV-vis to determine the concentration of DOX loaded in silica. The loaded silicas (becoming D1-5 samples) were washed with distilled water and dried in dark conditions (Figure 9c). The concentration of DOX in silica was calculated based on the molar extinction coefficient of DOX in PBS pH 7.4 (ε = 5498 M^−1^cm^−1^ at λ_max_ = 482 nm).

The encapsulation efficiency (EE%) and the loading capacity (LC) were calculated using the formulas:

(1) $$EE\left(\%\right)=\frac{W_{\left(drug\;in\;silica\right)}}{W_{\left(initial\;added\;drug\right)}}\times 100\;$$

(2) $$LC=\frac{W_{\left(drug\;in\;silica\right)}}{W_{\left(particles\right)}}$$

where *W* (*drug in silica*) is the amount of DOX in the MS sample calculated by the differences between the initial DOX amount and the DOX in the supernatant after the loading process, *W* (*initial added drug*) is the amount of the DOX in the stock DOX solution 0.1% used for loading experiments, and *W* (*particles*) is the total particles weight (mg). The calculated values for EE % and LC are shown in Table 7.

### 4.4. DOX Release Studies

Studies on the release of DOX were performed at different pH values: 1.5, 5, and 7.4. The concentration of DOX was calculated based on the molar extinction coefficients obtained by performing the calibration curves of DOX at pH 1.5 (ε = 11,324 M^−1^ cm^−1^, λ_max_ = 482 nm), pH 5 (ε = 8361 M^−1^ cm^−1^, λ_max_ = 482 nm), and pH 7.4 (ε = 5498 M^−1^ cm^−1^, λ_max_ = 482 nm). The DOX-loaded silica (10 mg/10 mL medium) was transferred into a dialysis membrane tubing (molecular weight cutoff 3.5 kDa), which then was immersed into PBS solution (50 mL) with pH 1.5, 5, and 7.4 at 37 °C with gentle shaking. At fixed time periods during 72 h, 2 mL of the release medium was removed and replaced with an equal volume of fresh PBS medium. The DOX release concentration at each predetermined interval of time was calculated based on UV-vis spectrophotometer measurements. The cumulative release (*CR*, %) (Equation (3)) in dependence on time was represented as mean values of three independent release experiments with standard deviation.

(3) $$CR\left(\%\right)=\frac{C_{DOX\;released}}{C_{DOX\;encapsulated}}\times 100\;$$

where *CR* is the cumulative release, *C_DOX released_* is the concentration of DOX released at different pH values, and *C_DOX encapsulated_* is the concentration of DOX encapsulated determined by UV-vis calibration curves.

The release of DOX was evaluated by the Higuchi (Equation (4)), Korsmeyer–Peppas (Equation (5)), and Peppas–Sahlin (Equation (6)) models, which have proven to fit best the experimental data [23]:

(4) $$M_{t}=K_{H}\times t^{1/2}$$

where $M_{t}$ is the amount of DOX released at $t^{1/2}$, and $K_{H}$ is the release rate constant.

(5) $$\frac{M_{i}}{M_{\infty }}=K\times t^{n}$$

(6) $$\frac{M_{t}}{M_{\infty }}=K_{1}t^{n}+K_{2}t^{2n}$$

where *M_i_* is the amount of DOX released at time *t* and *M_∞_* is the initial amount of DOX. *K*, *K*_1_ (assigned to the *diffusion process*), and *K*_2_ (assigned to the *relaxation process*, *case II transport*) are the release rate constants, and *n* is the exponent of release (diffusion exponent) used to evaluate the release mechanism.

### 4.5. Characterization Techniques

FT-IR spectra were registered with a Bruker Vertex 70 spectrometer, Ettlingen, Germany, operating in ATR mode with a ZnSe crystal in 600–4000 cm^−1^. The resolution of spectra was set to 4 cm^−1^ with an acquisition of 32 scans. The spectra have been processed by the device’s own software—OPUS 6.5. A number of steps were followed to obtain the spectral information: water and CO_2_ compensation, ATR-absorbance conversion, baseline correction, and normalization. The overlapped absorption bands were highlighted by the second derivative function from OPUS 6.5.

DLS analysis of MS samples, before and after DOX loading, was performed in quartz cuvettes at 25 °C using a Malvern Instruments Autosizer Lo-C 7032 Multi-8 correlator, Malvern Instruments, U.K., equipped with a HeNe laser, λ = 632.8 nm. The viscosity and the refractive index values were those of pure water. About 1 mg of each sample was dispersed in 5 mL of distilled water to prepare the stock solution. Before each registration, 0.5 mL of stock solution was diluted with 2 mL distilled water, and the mixture was ultrasonicated for 2 min with Elmasonic P equipment at 50% amplitude. For each registration, the data were acquired over40 s. Each sample was measured in three runs at a concentration of 0.005 wt% silica particles.

Zeta potential measurements were performed at a Zetasizer ZS equipment—Malvern Zetasizer IV, Malvern Instruments, U.K., in distilled water, at RT.

TEM (Hitachi HT7700, Hitachi High-Technologies Corporation, Tokyo, Japan) was used to investigate the morphology of the MSPs. The microscope operated at 100 kV in high contrast mode.

UV-vis spectrophotometer, Specord 210 Plus, Analytic Jena, was used to register the electronic spectra of DOX solution during the loading and release studies. The spectra were measured in 10 mm quartz cuvettes in the 200–700 nm spectral range using PBS solutions with pH 1.5, 5, and 7.4 as references.

CellTiter-Glo^®^ assay kit was used to perform the in vitro cell culture using a complete cell medium including aMEM, 10% FBS, and 1% antibiotic mixture in a humidified atmosphere with 5% CO_2_ at 37 °C. This procedure is based on the addition of the single reactive (CellTiter-Glo^®^ Reagent) directly in the cellgrowth medium, which will determine the cell lysis and the appearance of a luminescent signal, depending on the amount of ATP based on the total number of cells in the culture is estimated. Tryple was used for the detachment of the cells (5 × 10^3^ cell/well for HGF and 10 × 10^3^ cell/well for HeLa and MCF-7), PBS for washing operations, then the cells were centrifuged and seeded on 96-well opaque white plates for cell culture in a complete medium. The cell plates were incubated for 24 h at 37 °C (5% CO_2_), followed the next day by the addition of MS and DOX-loaded MS samples in a medium solution. The control sample was treated with the complete medium. The plates were incubated for 48 h, after which they were taken out and equilibrated for 30 min at RT, then 100 μL of CellTiter-Glo^®^ reagent was added, followed by another incubation of 15 min. The relative viability of the cells was evaluated using a plate reader (FLUOstar^®^ Omega, BMG-Labtech) based on the emitted light and calculated by the formula: Relative viability (%) = 100 − (RLU_p_ − RLU_blk_)/(RLU_c_ − RLU_blk_). The RLU_p_, RLU_c,_ and RLU_blk_ are the relative light units (RLUs) of the analyzed samples, control and blank, respectively. All the experiments were carried out in triplicate, being repeated three times for accuracy. The results of the cell viability were represented graphically with GraphPad Prism (version 8.00) for Windows.

Bioadhesion and mucoadhesion tests were performed using a TA.XT Plus^®^ texture analyzer (Stable Micro Systems, Godalming, UK) in order to measure the adhesion ability of the MS to cellulose membrane and porcine tissue mucosa (stomach, small and large intestine, and colon) in simulated physiological medium (37 °C, 250 r.p.m., PBS medium with pH 1.5, 7.4, or 8.5 depending on the adhesion substrate), for a predetermined contact time of 30 s. These properties are estimated by measuring the mechanical work and the adhesion force of the material tested against a synthetic or biological surface. The testing parameters for experimental registrations were: pre-test speed 1.00 mm/s; test speed 1.00 mm/s; post-test speed 10.00 mm/s; applied force 1.00 gf; return distance 10.00 mm; contact time 30.00 s; trigger force 5.00 gf. The experiments were performed in triplicate. The resulting adhesion parameters: the detachment force and the work of adhesion were calculated directly by TA.XT Plus texture analyzer software and were calculated as mean values with standard deviation.

The antimicrobial effect of MS samples was investigated against three species of fungi: *Aspergillus fumigates*—26906 ATTC, *Penicillium frequentans*—38858 ATTC, and *Fusarium*—60289 ATTC and two bacteria: *Bacillus* sp.—49342 ATTC and *Pseudomonas* sp.—19151 ATTC and compared with Kanamycin and Caspofungin as standard antibiotic and antifungal compounds. The strains were provided by the American Type Culture Collection (ATCC), USA. The strains were used as suspensions with a concentration of 10^−4^ μg/mL cultivated in sterile Petri dishes containing agar medium. The MS samples were placed in the Petri dishes prepared for analysis and incubated for 24 h at 37 °C. The diameter zone of inhibition was measured using a diagram of inhibition according to approved standards of the National Committee for Clinical Laboratory. The experiments were performed in triplicate, and the results are presented as mean values with standard deviation.

Nitrogen adsorption–desorption isotherms of MS particles were registered using AutosorbiQ Station1 equipment, Quanta Chrome Instruments, London, UK.

### 4.6. Statistical Analysis Was Used to Study the Differences among Means of the Obtained Data

A statistical tool based on variance analysis (ANOVA) was used to compare the experimental results. ANOVA analysis was conducted for a 5% confidence interval with the “α” parameter set to 0.05. *p* is the statistical probability used to certify a hypothesis against the observed data. This value evaluates the probability of obtaining the experimental results, assuming that the null hypothesis is true. The *p*-value < 0.05 is statistically significant. A *p*-value > 0.05 is not statistically significant and proves that the null hypothesis is evident.

## 5. Conclusions

MS particles functionalized with amino, hydroxyl, and methyl groups were synthesized by surfactant micelle-template and used as carriers for doxorubicin delivery.

The kinetic release models (Higuchi, Korsmeyer–Peppas, and Peppas–Sahlin) applied to the experimental data of DOX release showed that the least efficient release medium for the drug was PBS 7.4. The amino-functionalized MS exhibited lower values for DOX encapsulation and release with increased cumulative release at lower pH values (11.38% at pH 5 and 12.39% at pH 1.5). The hydroxyl-functionalized MS proved higher encapsulation efficiency (81%) but a lower release capacity (2.59%) at pH 1.5–7.4. Thus, pores and the surface functionality of MS are suggested to be responsible for the lower release of DOX. MS particles and the corresponding DOX-loaded MS samples induced cytotoxicity at concentrations higher than 30 μg/mL on normal (HGF) and IC_50_ between 10.84 ± 0.1 μg/mL (D3 on MCF-7) and 136.5 ± 0.2 μg/mL (D1 on HeLa). At higher concentrations, all samples exhibited increased cytotoxicity, with selectivity on MCF-7 cells. The particular studies (the influence on selected cultures of fungi and bacteria and bio/mucoadhesion tests) demonstrated the ability of the functionalized MS materials to act as antimicrobial and mucoadhesive agents. The highest antimicrobial activity, comparable with that of commercial antibiotic Kanamycin and antifungal Caspofungin used as standards, and increased mucoadhesivity were found for the MS functionalized with amino- and hydroxyl groups. These functional groups can be used as an advantage to promote prolonged contact with the mucosa through specific interactions with the mucus layer.

## Figures and Tables

**Table 1:** MS particle characteristics before and after DOX loading.

Sample	M1	D1	M2	D2	M3	D3	M4	D4	M5	D5
Size DLS (nm)	825.2 ± 0.3–1280.3 ± 1.2	615.3 ± 0.4–825.1 ± 2.2	712.4 ± 1.6–955.2 ± 2.5	396.5 ± 4.5–712.4 ± 0.9	396.2 ± 4.2–531.6 ± 2.2	1484.2 ± 12.3–2305.8 ± 23.5	295.3 ± 0.8–825.7 ± 0.4	396.4 ± 2.2–712.4 ± 0.9	255.2 ± 1.1–458.7 ± 0.6	342.4 ± 2.2–615.6 ± 2.5
PDI *	0.216 ± 0.11	0.305 ± 0.08	0.546 ± 0.12	0.614 ± 0.04	0.478 ± 0.13	0.397 ± 0.09	0.641 ± 0.21	0.611 ± 0.13	0.572 ± 0.15	0.603 ± 0.22
Size by TEM (nm)	>500	>500	>500	>500	200–400	200	200–400	200	200–400	200
Zeta potential (mV)	2.98 ± 0.62	11.10 ± 3.72	38.00 ± 5.54	−6.10 ± 1.47	−9.29 ± 2.88	−11.8 ± 0.47	−16.0 ± 2.7	−10.7 ± 1.1	−10.8 ± 1.31	−9.81 ± 1.82

* Polydispersity index. M1, M2, M3, M4, and M5 denoted the initial MS samples before DOX loading, while D1, D2, D3, D4, and D5 denoted the DOX-loading MS samples. All data are shown as the mean with standard deviation (SD) from at least three independent experiments. Statistical evaluation of the variance differences was conducted using ANOVA at significance levels of *p* < 0.05.

**Table 2:** Kinetic parameters of DOX release process from MS samples.

Kinetic Models									
Sample	Higuchi		Korsmeyer–Peppas			Peppas–Sahlin			
pH 7.4									
	*K_H_*	R^2^	*n*	*K*	R^2^	*n*	*K*_1_	*K*_2_	R^2^
D1	0.1495	0.9814	0.4238	0.2718	0.9858	0.2479	0.2340	0.1207	0.9842
D2	0.1517	0.9573	0.3907	0.3570	0.9676	0.2360	0.3491	0.1332	0.9652
D3	0.0349	0.9550	0.5402	0.0304	0.9814	0.5266	0.0281	0.0011	0.9814
D4	0.0338	0.9726	0.6346	0.0098	0.9511	0.6330	0.0099	1.35 × 10^−5^	0.9839
D5	0.1672	0.9647	0.5485	0.1141	0.9633	0.3337	0.1670	0.0325	0.9597
pH 5									
	*K_H_*	R^2^	*n*	*K*	R^2^	*n*	*K*_1_	*K*_2_	R^2^
D1	0.1958	0.9866	0.4314	0.3352	0.9890	0.2667	0.3735	0.1041	0.9879
D2	0.1739	0.9822	0.4498	0.2576	0.9852	0.2835	0.3353	0.0662	0.9830
D3	0.0406	0.9781	0.3657	0.1150	0.9828	0.1633	0.4368	0.0287	0.9828
D4	0.0383	0.9927	0.3674	0.0969	0.9948	0.2139	0.0093	0.0653	0.9932
D5	0.1541	0.9570	0.8301	0.0114	0.9752	0.8281	0.0101	1.58 × 10^−6^	0.9660
pH 1.5									
	*K_H_*	R^2^	*n*	*K*	R^2^	*n*	*K*_1_	*K*_2_	R^2^
D1	0.2091	0.9927	0.3961	0.4720	0.9974	0.2599	0.6213	0.0955	0.9969
D2	0.1858	0.9907	0.4180	0.3531	0.9951	0.2459	0.2332	0.1620	0.9935
D3	0.0413	0.9017	0.2542	0.2826	0.9579	0.1567	0.3676	0.0663	0.9543
D4	0.0458	0.9728	0.2863	0.2433	0.9888	0.1819	0.2715	0.0663	0.9890
D5	0.1523	0.9666	0.7957	0.0148	0.9802	0.7921	0.0148	6.17 × 10^−7^	0.9561

**Table 3:** Results of CellTiter-Glo assay presented as IC_50_ (μg/mL) values obtained after 48 h of incubation.

IC_50_ (μg/mL)				SI^a^	
MS Samples	HeLa	MCF-7	HGF	SI_1_^b^	SI_2_^c^
D1	136.5 ± 0.2 (0.90 ± 0.2 μg/mL DOX)	73.98 ± 0.1 (0.49 ± 0.2 μg/mL DOX)	60.06 ± 0.2 (0.40 ± 0.2 μg/mL DOX)	0.44	0.81
D3	18.95 ± 0.2 (0.59 ± 0.1 μg/mL DOX)	10.84 ± 0.2 (0.33 ± 0.1 μg/mL DOX)	-	-	-
D5	43.66 ± 0.3 (1.56 ± 0.1 μg/mL DOX)	35.54 ± 0.3 (1.27 ± 0.1 μg/mL DOX)	24.24 ± 0.2 (0.86 ± 0.2 μg/mL DOX)	0.55	0.68

SI^a^—selectivity index; SI_1_^b^ = IC_50_ (HGF)/IC_50_ (HeLa); SI_2_^c^ = IC_50_ (HGF)/IC_50_ (MCF-7). All data are presented as the mean with standard deviation from three independent experiments. Statistical evaluation of differences was conducted using ANOVA at significance levels of *p* < 0.05.

**Table 4:** The mucoadhesion parameters of the MS samples on different segments of tissue mucosae.

Sample	Mucoadhesion on Different Tissue Mucosae							
	Detachment Force (N) (×10^−2^)				Work of Adhesion (mJ) (×10^−2^)			
	Stomach	Small Intestine	Large Intestine	Colon	Stomach	Small Intestine	Large Intestine	Colon
M1	4.34 ± 0.07	7.09 ± 0.39	4.61 ± 0.19	8.82 ± 1.28	0.99 ± 0.39	0.62 ± 0.37	0.55 ± 0.05	1.34 ± 0.88
M2	3.85 ± 0.05	16.50 ± 2.87	5.19 ± 0.19	8.79 ± 2.16	0.85 ± 0.04	3.17 ± 0.86	0.80 ± 0.02	1.20 ± 0.71
M3	4.02 ± 0.16	5.88 ± 0.19	6.01 ± 0.28	5.94 ± 0.24	0.80 ± 0.04	0.69 ± 0.02	0.97 ± 0.05	0.56 ± 0.10
M4	3.98 ± 0.28	9.08 ± 2.33	5.32 ± 0.14	8.17 ± 1.24	0.72 ± 0.02	2.57 ± 1.11	0.96 ± 0.03	1.08 ± 0.98
M5	4.15 ± 0.03	5.68 ± 0.01	4.61 ± 0.17	10.42 ± 0.55	0.86 ± 0.04	0.38 ± 0.12	0.80 ± 0.006	3.39 ± 0.25

All data are provided as the mean with standard deviation from three independent experiments. Statistical evaluation of the variance differences was conducted using ANOVA at significance levels of *p* < 0.05.

**Table 5:** The antimicrobial activity of the MS samples.

Sample	MIC ^a^ (µg/mL)				
	Fungi			Bacteria	
	*Aspergillus* *fumigatus*	*Penicillium* *frequentans*	*Fusarium*	*Bacillus* sp.	*Pseudomonas* sp.
M1	0.38 ± 0.17	0.38 ± 0.11	0.38 ± 0.09	2.00 ± 0.02	2.00 ± 0.22
M3	0.75 ± 0.09	0.75 ± 0.12	0.75 ± 0.07	3.00 ± 1.11	3.00 ± 1.12
M5	>32	>32	>32	>256	>256
Caspofungin ^b^	0.20 ± 0.02	0.20 ± 0.02	0.20 ± 0.02	-	-
Kanamycin ^b^	-	-	-	3.00 ± 0.02	3.00 ± 0.02

^a^—minimum inhibitory concentration; ^b^—standard antifungal and antibacterial compounds. All data are shown as the mean with standard deviation from three independent experiments. Statistical evaluation of the variance differences was conducted using ANOVA at significance levels of *p* < 0.05.

**Table 6:** The main characteristics of the organo-modified and/or functionalized silica particles.

Sample	M1	M2	M3	M4	M5
Surface group	-(CH_2_)_3_NH_2_	-(CH_2_)_3_NH_2_	-OH	-OH	-CH_3_
Pore size (nm)	3.33 ± 0.01	3.02 ± 0.02	3.06 ± 0.01	3.04 ± 0.01	2.49 ± 0.06
BET area (m^2^/g)	722.08 ± 7.32	621.11 ± 15.24	1001.01 ± 12.43	936.20 ± 4.22	943.13 ± 0.74

**Table 7:** Loading of DOX in terms of EE % and LC in the MS particles.

Samples	EE, %	LC μg/mg
M1	16.75 ± 0.04	6.70 ± 0.22
M2	14.13 ± 0.11	5.65 ± 0.09
M3	81.80 ± 0.12	32.70 ± 0.24
M4	78.74 ± 0.01	31.49 ± 0.11
M5	90.00 ± 0.02	36.00 ± 0.03

## Data Availability

Not applicable.

## Supplementary Materials

The following supporting information can be downloaded at: https://www.mdpi.com/article/10.3390/ijms241914687/s1.
