# Supplementary material for: Mucoadhesive Mesoporous Silica Particles as Versatile Carriers for Doxorubicin Delivery in Cancer Therapy
Source: Int J Mol Sci. 2023 Sep 28;24(19):14687. doi: 10.3390/ijms241914687 (PMC10572865; doi:10.3390/ijms241914687)
Supplement: Supplementary file 1 [file ijms-24-14687-s001.zip › ijms-2627340-supplementary.pdf]

Supplementary Material

Mucoadhesive Mesoporous Silica Particles as Versatile Carriers for Doxorubicin Delivery in Cancer Therapy

Mirela-Fernanda Zaltariov [1,*], Bianca-Iulia Ciubotaru [1], Alina Ghilan [2], Dragos Peptanariu [3], Maria Ignat [1,4], Mihail Iacob [1], Nicoleta Vornicu [5] and Maria Cazacu [1,*]

[1] Department of Inorganic Polymers, "Petru Poni" Institute of Macromolecular Chemistry, Aleea Gr. Ghica Voda 41 A, 700487 Iasi, Romania; ciubotaru.bianca@icmpp.ro (B.-I.C.); ignat.maria@icmpp.ro (M.I.); iacob.mihai@icmpp.ro (M.I.)
[2] Department of Natural Polymers, Bioactive and Biocompatible Materials, "Petru Poni" Institute of Macromolecular Chemistry, Aleea Gr. Ghica Voda 41 A, 700487 Iasi, Romania; diaconu.alina@icmpp.ro
[3] Centre of Advanced Research in Bionanoconjugates and Biopolymers, "Petru Poni" Institute of Macromolecular Chemistry, Aleea Gr. Ghica Voda 41 A, 700487 Iasi, Romania; peptanariu.dragos@icmpp.ro
[4] Department of Chemistry, "Alexandru Ioan Cuza" University of Iasi, 700506 Iasi, Romania
[5] Metropolitan Center of Research T.A.B.O.R, The Metropolitanate of Moldavia and Bukovina, 700497 Iasi, Romania; cmctaboriasi@yahoo.com
* Correspondence: zaltariov.mirela@icmpp.ro (M.-F.Z.); mcazacu@icmpp.ro (M.C.)

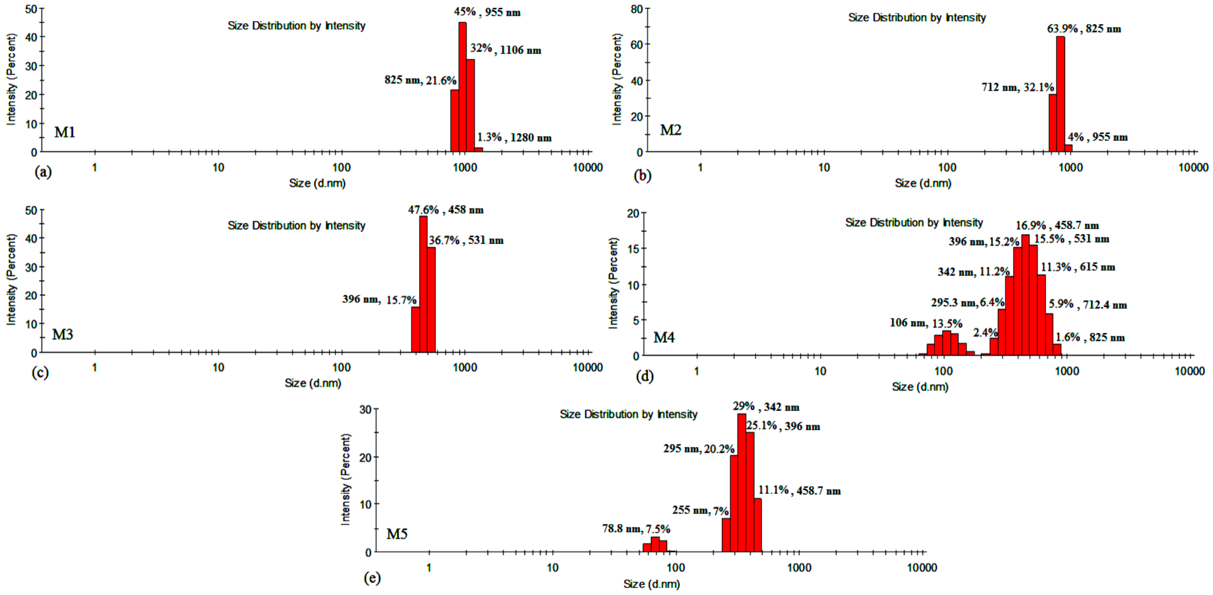

Figure S1. The size distribution of the MS particles (nm) determined by DLS: M1 (a), M2 (b), M3 (c), M4 (d), M5 (e).

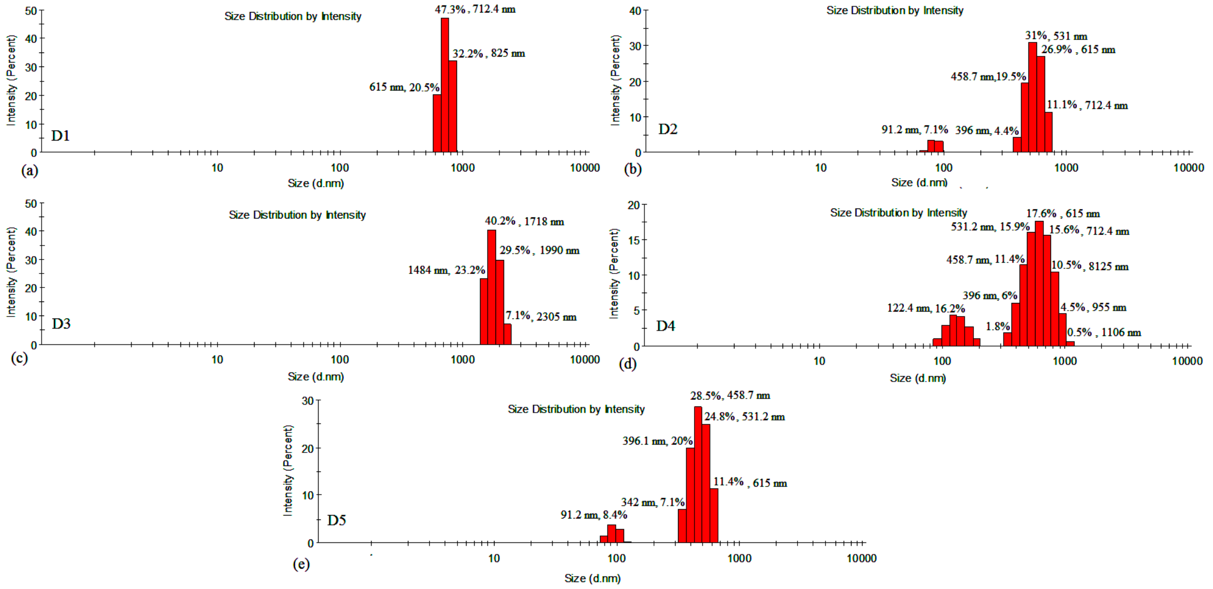

Figure S2. The size distribution of the MS particles (nm) determined by DLS: D1 (a), D2 (b), D3 (c), D4 (d), D5 (e).

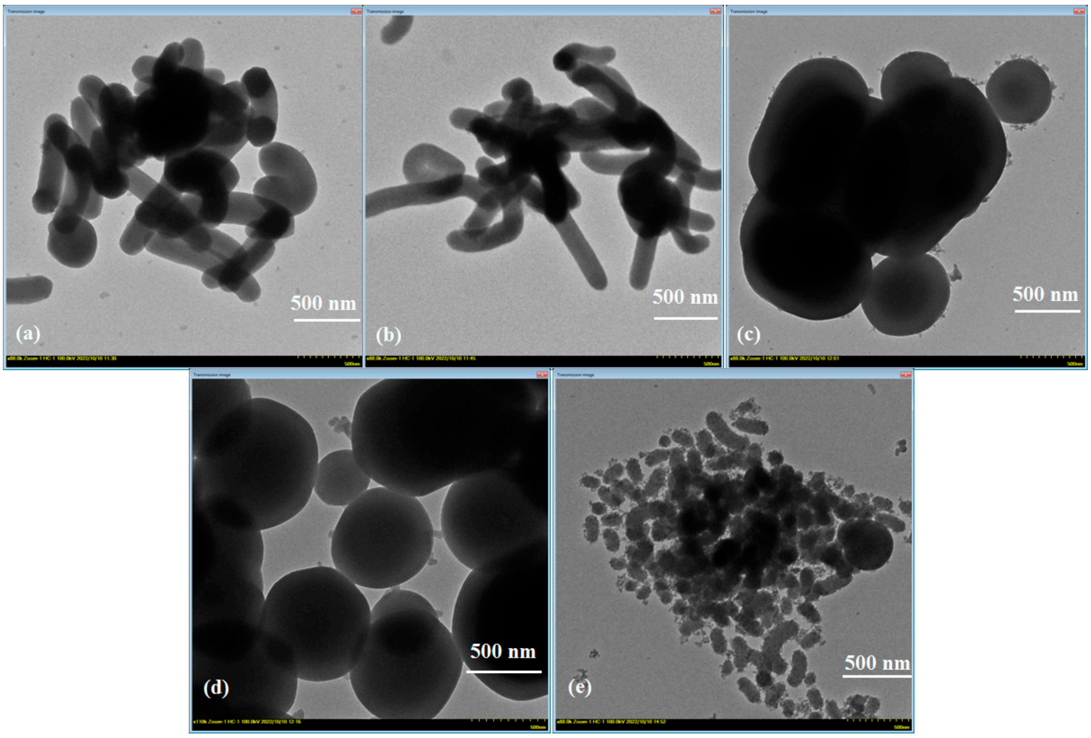

Figure S3. TEM images of MS samples before DOX loading: M1 (a), M2 (b), M3 (c), M4 (d), M5 (e).

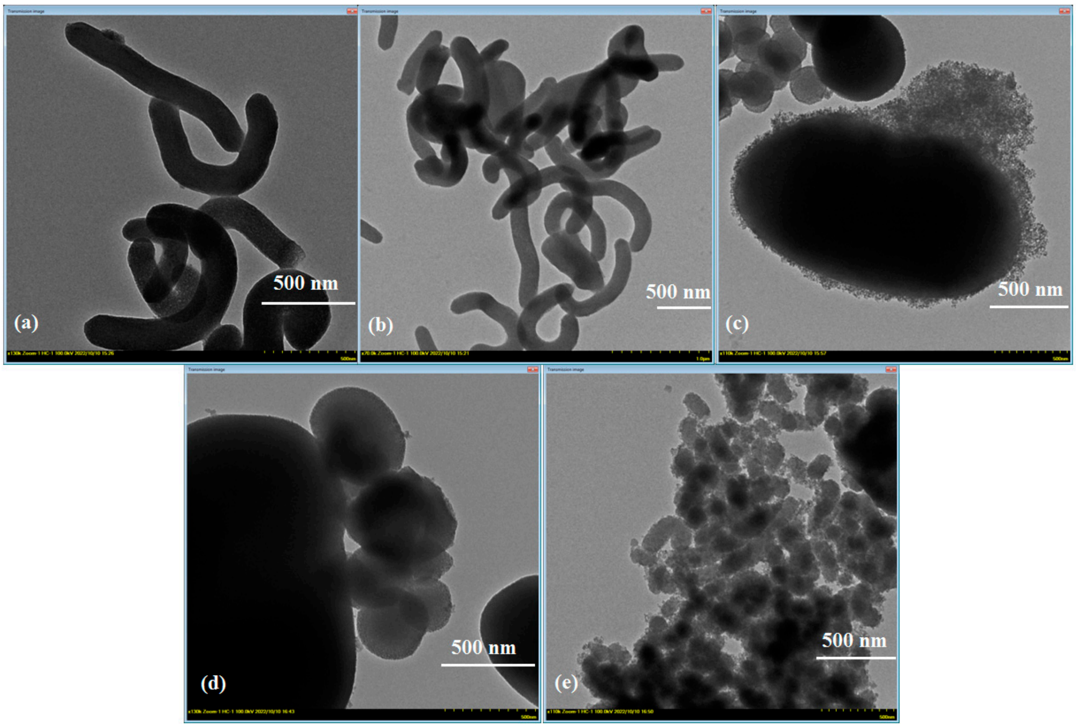

Figure S4. TEM images of DOX-loaded MS particles: D1 (a), D2 (b), D3 (c), D4 (d), D5 (e).

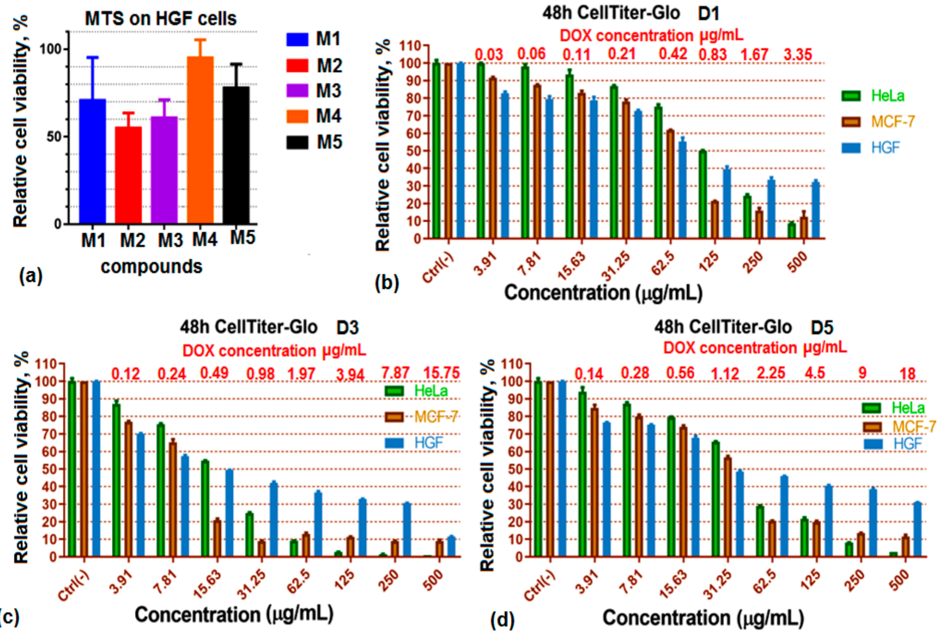

Figure S5. Cytotoxicity of the MS samples, before DOX loading (a) and after encapsulation of DOX: D1 (b), D3 (c) and D5 (d).

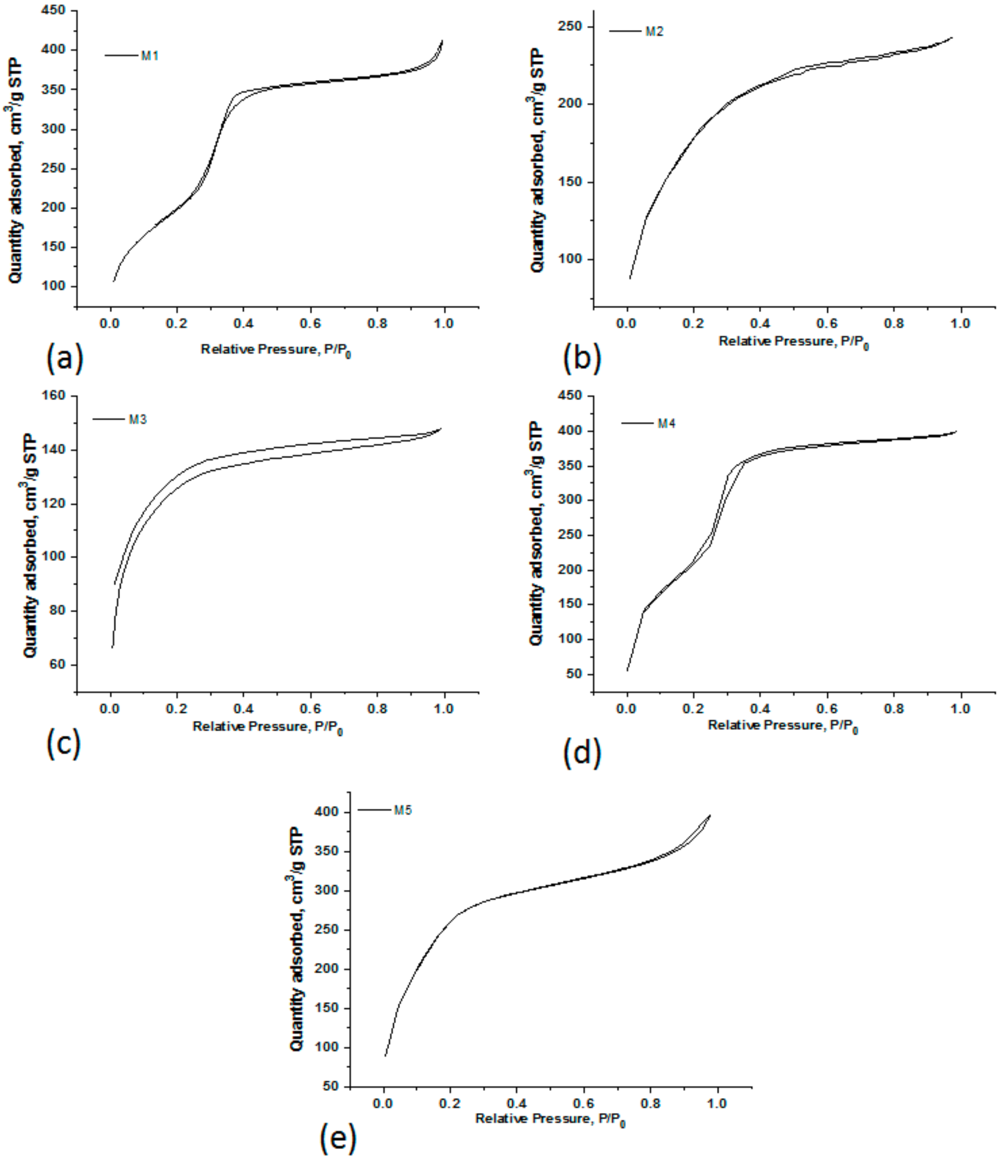

Figure S6. N_2 isotherms of M1 (a), M2 (b), M3 (c), M4 (d) and M5 (e) MS samples.
